# Lipid Profile in Relation to Anthropometric Measurements among College Male Students in Riyadh, Saudi Arabia: A Cross-Sectional Study

**Published:** 2011-06

**Authors:** Abdul Rahman Al-Ajlan

**Affiliations:** *Department of Clinical Laboratory Sciences, Riyadh College of Health Sciences, King Saud University, Saudi Arabia*

**Keywords:** obesity, waist circumference, hip circumference, overweight, BMI, total cholesterol, HDL, LDL, TG, dyslipidemia

## Abstract

**Background:**

Anthropometric measurements can easily reflect any changes in the lipid concentration in the human body.

**Objectives:**

The present work is aimed at studying lipid profile and its relation to anthropometric measurements in college males from Riyadh, Saudi Arabia.

**Subjects and methods:**

This study was conducted from September 2006 to December 2008. 333 students aged 18-35 years of Riyadh College of Health Science - male section - participated in the study. Anthropometric measurements including weight, height, waist and hip circumferences were measured. Body Mass Index (BMI) was calculated. Fasting blood sugar and lipid profile including total cholesterol (TC), Low density lipoprotein cholesterol (LDL), high density lipoprotein cholesterol (HDL) and triglycerides (TG) were estimated. Socio-demographic data were collected from a questionnaire.

**Results:**

Mean TC level was 4.227 ± 0.869 mmol/l, while for LDL, HDL and TG were 2.57 ± 0.724, 1.360 ± 0.545 and 1.385 ± 0.731 mmol/l, respectively. Mean TC level did not differ significantly across weight groups except among obese patients. Mean HDL, LDL and TG did not differ significantly among different groups at 5% level of significance. There was positive, statistically non-significant correlation between age and BMI. The correlation between age and all lipid parameters were statistically non-significant. There was positive correlation between BMI and TC and LDL, while there was a negative correlation between BMI and HDL. There was no correlation between BMI and triglycerides.

**Conclusion:**

BMI, waist and hip circumferences all increase with age. The level of TC, LDL and TG go high with increase in age and BMI.

## INTRODUCTION

The associations between overweight and many diseases have been established. Body-fat distribution could possibly identify subjects with the highest risk of disturbed lipid profile and hypertension. Disturbed lipid profile has always been associated with cardiovascular diseases. Anthropometric measurements can easily reflect any changes in the lipid concentration in the human body (1).

Obesity is a worldwide health problem. It is associated with excessive fat accumulation in the body to the extent that health and well being are adversely affected. With changing food habits and sedentary lifestyles, the prevalence of obesity has increased markedly in Western countries faster than the developing ones (2). It was reported that 30% of the population in the United States in 1995 were overweight. Obesity may increase the risk of many diseases such as diabetes, atherosclerosis, hypertension, hyperlipidemia, gall bladder diseases and cardiovascular diseases (3).

Intra-abdominal fat has been identified as being the most clinically relevant type of fat in humans. Increased level of LDL, high TC, and low levels of HDL are frequently observed in combination with hypertriglyceridemia (4). Body mass index, waist and hip circumferences were found to be useful anthropometric predictors for cardiovascular risk (5). A comprehensive nutritional evaluation is also important in these cases. It should involve the subject and his family members. Even adolescents in charge of their own meals should have family members involved in parts of the assessment and counselling (6). The dietary assessment should include: usual food choices at home, sources of saturated fat and cholesterol, and food choices away from home including those from vending machines, snack, and school lunch (7).

Adolescents with high TC or LDL may have a genetic disorder of lipid metabolism such as familial hypercholesterolemia. Those with homozygous chromosomes forms can experience myocardial infarction or other events in early age. Familial hypercholesterolemia is often diagnosed in adolescence and is characterized by high LDL levels that can be refractory to dietary treatment (8). Other causes of dyslipidemia include: anabolic steroid use, anorexia nervosa, cigarette smoking, diabetes, glycogen storage diseases, hypothyroidism, liver disease and such medications like corticosteroids, anticonvulsants and certain oral contraceptives. Other causes like overweight and obesity, renal disease, therapeutic diet (ketogenic and high carbohydrate diet) and transplant (bone marrow, heart, kidney, or liver) may also cause dyslipidemia (9).

In adults, high LDL is strongly associated with a higher risk of coronary heart disease (CHD) while high HDL is usually protective. Lowering lipids through dietary or pharmacological therapy has been shown to decrease the incidence of atherosclerotic events. The extent of abnormal lipids and other cardiovascular risk factors in adolescence is related to the severity of atherosclerosis (10).

Encouraging omega-3-fatty acid consumption, increasing dietary fibre intake, fruits, vegetables, cereals, oats, whole grains and legumes are good sources of soluble fibre. Antioxidant food sources - carotenoids and vitamins C and E - may lower CHD risk. Recommended antioxidant-rich foods such as whole grains, citrus fruits, melons, berries and dark orange/yellow or leafy green vegetables act as supplements (11, 12).

Recent recommendations stress that weight management includes optimizing LDL, HDL and TG levels. Increased physical activity, quitting smoking, follows up and monitoring are essential. Selective lipid screening is recommended when we have a strong family history or two or more CHD risk factors. Increased physical activity can also increase muscles bulk and improve tissue response to insulin without significant weight loss (13, 14).

## AIM OF THE STUDY

Investigation of the relationship between anthropometric measurements and lipid profile among college male students from Riyadh, Saudi Arabia.

### Subjects and methods

**Study design:** a cross-sectional study.

**Study population:** all students of Riyadh College of Health Science - male section (333 subjects), studying or registered from September 2006 to December 2008 were the target population of the study. Their age bracket was 18-35 years. A written consent was signed by each participant after full explanation of the procedure of the study. All students had the right to withdraw at any time during the study without any explanation and without any threat of punishment. They were told that all the data will be confidential and will be used only for research purposes.

**Methods of the study:** All subjects answered a pre-designed and pre-tested questionnaire during or after college hours. The questionnaire included socio-demographic data, present, past and family history of any medical condition, data about physical activity and dietary habits. Results concerning physical activity and dietary habits were not included in the study. At the end of the session, anthropometric measurements were taken. All participants were asked not to eat after 10 pm. the evening before test. And those studying in afternoon shift were asked to fast at least for 12 hours next day. The following day, 5 ml venous blood sample was collected from each participant for laboratory analysis.

**Anthropometric measurements:** weight, height, waist circumference and hip circumference were measured after completing the questionnaire sheet.

**Weight:** was measured to the nearest 0.1 kg in light clothing and standing barefoot using a well calibrated balance scale, which is a part of the body composition analyzer (model 3P7044, Webb city, MO, USA).

**Height:** was measured to the nearest 0.5 cm using a wooden meter fixed on the wall while the subject was standing relaxed, barefoot and heels together touching the wall.

**Waist and hip circumference (WC):** were measured twice to the nearest 0.5 cm, with a flexible but non-elastic measuring tape. Waist circumference was measured at level of the natural waist (the narrowest part of the torso) or one finger width below the umbilicus. Hip circumference was measured at the maximum circumference of the buttocks posteriorly and the symphysis pubis anteriorly, in a horizontal plane (15).

**BMI:** was calculated by dividing the body weight (in kilograms) by the height (in meters squared) (16).

### Laboratory investigations

Venous blood was drawn for biochemical examination which included fasting blood sugar and lipid profile. TC, (HDL-C and TG were estimated directly while LDL-C was calculated using Friedewald formula (17). The initial venous blood sample was collected in plain tube (red top tube) for serum lipid and into fluoride oxalate tube for fasting glucose measurements. Collected blood samples were sent to King Saud Medical Complex Research Laboratory for analysis. Lipid profile was performed using enzymatic methods on the Biosystems A25 manufactured in Spain. TC concentration was estimated using the cholesterol oxidase/peroxidase method (CV 1.9% at 3.68 mmol/l and 1.5% at 6.27 mmol/l), and glycerol kinase method (CV 2.8% at 0.50 mmol/l and/triglyceride using the lipase 1.6% at 2.34 mmol/l). HDL-C was measured (CV 2.25% at 0.89 mmol/l and 3.5% at 1.12 mmol/l) after precipitation of very low density lipoprotein (VLDL) and LDL by polyethylene glycol PEG 6000. LDL-C was calculated using Friedewald equation. Fasting blood sugar was estimated using glucose oxidase/peroxidase method (CV 1.3% at 4.66 mmol/l and 1.5% at 14.43 mmol/l).

### Statistical analysis

Data were collected, presented and statistically analyzed using “SPSS statistical package for Windows version 15”. Mean, standard deviation and Student’s “t”-test were used to compare quantitative data. Frequencies and chi-square test were used to compare qualitative data. Correlation coefficient was used to compare continuous variables. The level of significance used was 5% level.

## RESULTS

**Figure 1:**
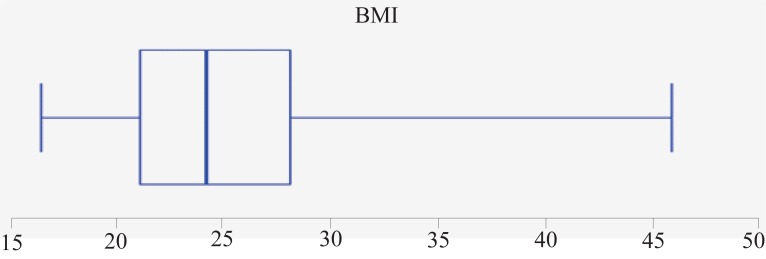
Distribution of participants according to BMI.

**Figure 2:**
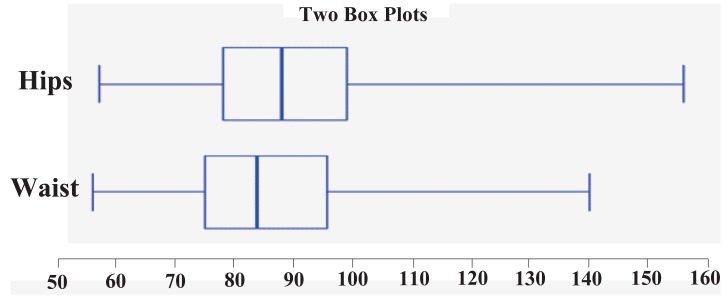
Distribution of participants according to HC and WC.

Table 1, Figure 1 and Figure 2 show characteristics of studied subjects. BMI of studied subjects ranged from 16.37 to 45.97 with a mean of 25.26 ± 5.86. WC ranged from 56 to 140 cm with a mean of 85.85 ± 14.28 cm. HC ranged from 57 to 156 cm with a mean of 89.96 ± 15.17 cm. Mean age of subjects was 20.727 ± 2.947 years. Mean TC was 4.227 ± 0.869 mmol/l, 2.57 ± 0.724, 1.36 ± 0.545, and 1.385 ± 0.731 for LDL, HDL, and TG respectively.

**Table 1:** Age, anthropometric measurements and lipid profile of studied subjects

Characteristics	Mean	Standard deviation

Age (years)	20.727	2.947
Height (cm)	170.73	5.57
Weight (Kg)	73.721	17.77
BMI W/(H in m)^2^	25.258	5.856
Waist circumference (cm)	85.847	14.279
Hip circumference (cm)	89.961	15.167
Total cholesterol (mmol/l)	4.227	0.869
LDL (mmol/l)	2.570	0.724
HDL (mmol/l)	1.360	0.545
Triglycerides (mmol/l)	1.385	0.731
Systolic blood pressure	113.601	6.004
Diastolic blood pressure	74.895	5.548
Fasting blood sugar (mmlo/dl)	4.984	0.889

Table 2 shows lipid profile among study subjects in relation to BMIMean TC level did not differ significantly between groups except among obese subjects in whom it was significantly higher (4.46 ± 0.96 mmol/l; *p*=0.03). Mean HDL was higher significantly among underweight students (1.52 ± 0.89 mmol/l; *p*=0.04). Mean LDL and triglyceride did not differ significantly among different groups.

**Table 2:** Lipid profile in relation to body mass index

Lipid profile	Normal weight n=129 (38.74)	Underweight n=59 (17.72)	Overweight n=88 (26.43)	Obese n=48 (14.41)	Severe obese n=9 (2.7)

TC	4.14 (0.81)	4.07 (0.86)	4.34 (0.89)	4.46 (0.96)	4.17 (0.81)
“t” (p)		0.53 (0.59)	1.72 (0.09)	**2.22 (0.03)**	0.11 (0.91)
HDL	1.33 (0.39)	1.52 (0.89)	1.30 (0.52)	1.36 (0.40)	1.29 (0.26)
“t” (p)		**2.04 (0.04)**	0.49 (0.63)	0.45 (0.65)	0.3 (0.76)
LDL	2.58 (0.7)	2.46 (0.61)	2.59 (0.77)	2.61 (0.83)	2.72 (0.77)
“t” (p)		1.13 (0.26)	0.1 (0.92)	0.24 (0.81)	0.58 (0.57)
Triglycerides	1.44 (0.89)	1.26 (0.52)	1.41 (0.66)	1.38 (0.6)	1.17 (0.62)
“t” (p)		1.44 (0.15)	0.27 (0.79)	0.43 (0.67)	0.89 (0.37)

**Figure 3:**
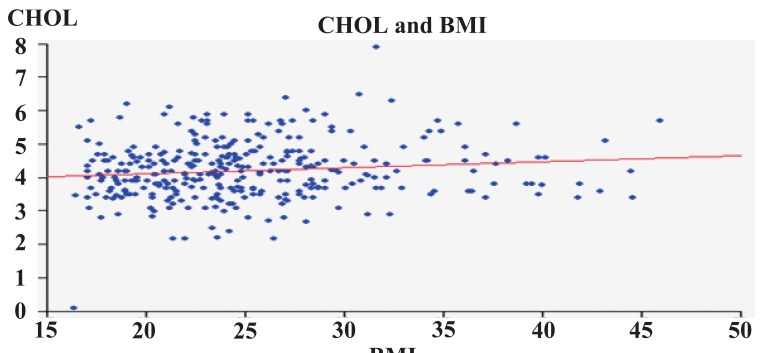
TC in relation to BMI.

**Figure 4:**
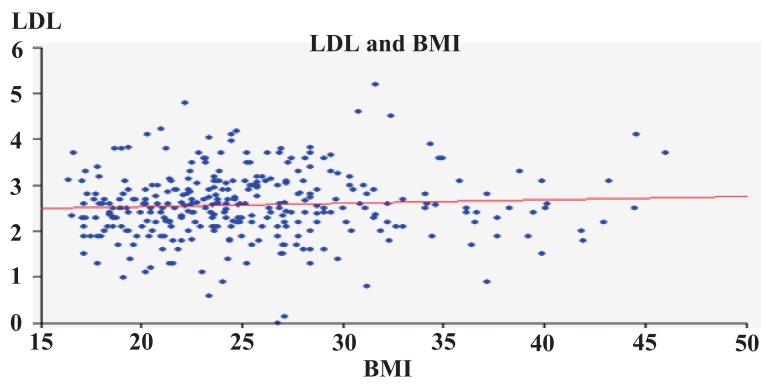
LDL in relation to BMI.

**Figure 5:**
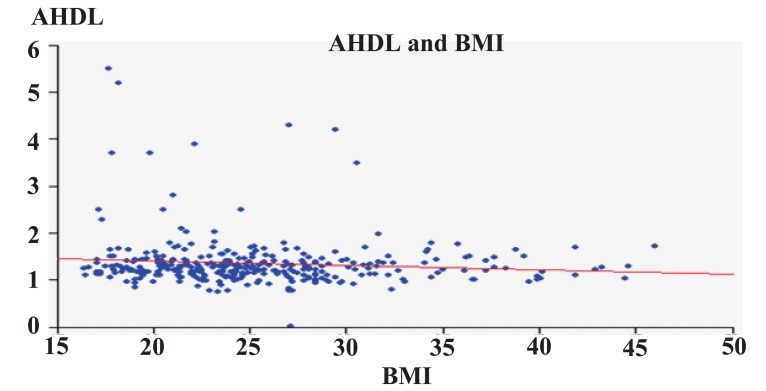
HDL in relation to BMI.

**Figure 6:**
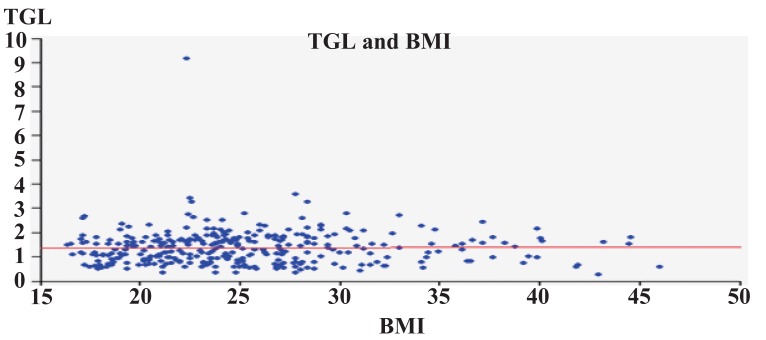
TG in relation to BMI.

Figures 3-6 show correlation between BMI and TC, LDL, HDL, and triglycerides. There was positive correlation between BMI and TC and LDL. While there was a negative correlation between BMI and HDL, there was no correlation between BMI and triglycerides.

## DISCUSSION

One of the most common problems related to lifestyle today is being overweight. Severe overweight or obesity is a key risk factor in the development of many chronic diseases such as heart and respiratory diseases, non-insulin-dependent diabetes mellitus or Type 2 diabetes, hypertension and some cancers, as well as early death. New scientific studies and data from life insurance companies have shown that the health risks of excessive body fat are associated with relatively small increases in body weight, not just with marked obesity (18). Obesity and overweight are serious problems that pose a huge and growing financial burden on public resources.

Evaluations of the effects of excess weight on health should consider the distribution of body fat as well as the amount of adipose tissue. Abdominal fat has been associated with insulin resistance (19), hyperlipidemia, hypertension (20), certain types of cancer (21) and osteoporosis (22).

World Health Organization recommends measurement of the BMI as a universal criterion of overweight (≥25) and obesity (≥30) while measures of abdominal fat distribution such as WC or waist-to-hip ratio (WHR) are also advised (23). There is evidence to support the use of BMI in risk assessment since it provides a more accurate measure of total body fat compared with the assessment of body weight alone. BMI does not however, distinguish fat from muscle. Excess abdominal fat is an important, independent risk factor for disease. Men who have waist circumference greater than 40 inches and women who have waist circumference greater than 35 inches are at higher risk for developing diabetes, elevated cholesterol levels, hypertension, and CVD because of excess of abdominal fat (24). WC measurement is particularly useful in people who are categorized as normal or overweight in terms of BMI. For individuals with BMI>35, waist circumference adds little to the predictive power of the disease risk. A high WC is associated with an increased risk of type 2 diabetes, dyslipidemia, hypertension, and CVD in patients with a BMI between 25 and 34.9 kg/m.

Evidence suggests that the prevalence of overweight and obesity is rising dramatically worldwide and that the problem appears to be increasing rapidly in children as well as in adults. In Korea and other Asian countries, more and more of the population is becoming obese and many people may be under increasing threat of developing metabolic syndrome. The third national health and nutrition survey conducted by the Korean Ministry of Health and Welfare in 2001 announced that the overall prevalence of obesity in Korean adults was 30.6% (32.4% in men, 29.4% in women) (25).

High prevalence of obesity was noted in our study compared to what has been noted in other urban studies on obesity (26, 27). Mean value of the BMI recorded in the present study was 25.26 ± 5.86 kg/m^2^. This is akin to data derived from migrant Indians to the USA (28). This probably was because our study group was mostly from higher socio-economic strata and hence was not a true representative of the population. The developments of the Saudi economy in recent decades and the consequent social and cultural changes have altered dietary habits in this country. These changes are characterized by a decrease in consumption of grain products, green vegetables and legumes, together with an increase in the consumption of meat, potatoes, fruit, fat and dairy products (29, 30).

The most comprehensive data on the prevalence of obesity worldwide are those of MONICA project (Monitoring of Trends and Determinants in Cardiovascular Diseases Study) of the World Health Organization (31). The data shows that prevalence of obesity in most European countries has increased by about 10-40% in the past 10 years, ranging from 10-20% in men and 10-25% in women (32). The most alarming increase has been observed in United Kingdom, where nearly two thirds of adult men are overweight or obese (33).

Mean values for weight, height and BMI in our sample were similar to those described in a study of Spain (34). Compared to the reference values for a central European population (35, 36) mean height for the Saudi population was lower, while weight and BMI was generally higher.

The prevalence of obesity in our sample was similar to that in other parts of Saudi Arabia (37), in the province of Latina (Italy) (38), the United States (39), England (for men) (40) and Austria (41). However, the values we found were higher than in some European countries such as Switzerland (36), France (42), Naples (Italy) (38), Germany (43), Finland (44) and Sweden (45, 46).

Although the distribution of obesity has been widely documented, the distribution of WC and HC values in different countries is less well studied (44). Mean WC and HC values observed in the population we studied were similar to the mean values observed in populations that took part in the WHO MONICA European Project (47).

Cholesterol is a fat-like substance made by the body. It is used in the production of bile acids, steroid hormones, Vitamin E, and cell membranes. Dietary cholesterol is found in animal foods such as organ meats, egg yolks, other meats and poultry. Cholesterol levels can have a major impact on risk for heart disease. It is recommended that you maintain a low LDL-C (bad cholesterol) and a high HDLC (good cholesterol). According to the National Cholesterol Education Project, lowering LDL-C in moderate to high- risk people can lead to a reduction in cardiovascular events (48).

In Europe, mean values for plasma TC were lower than the values for the populations analyzed in the WHO MONICA European Project, with the exception of Poland, Russia and Sweden (31), and slightly higher than the values reported for the USA (49). Our mean HDL-C values were higher than those found for the adult population of England (23), the USA (49) and Brazil (50) adult male population in Germany (43) and France (49).

Although obesity has been associated with dyslipidemia (51), we found no significant association in lipid profile between normal and overweight persons. In fact, all mean values for all three subgroups were within normal range.

In the present study, even though BMI correlated with TC and LDL-C levels, it did not correlate with elevated TG and HDL-C levels. BMI has been widely used as an indicator of total adiposity; its limitations are clearly recognized by its dependence on race (Asians having large percentages of body fat at low BMI values), and age. As compared to BMI, WC and WHR have been used as surrogates of body fat centralization. The strength of association of WHR and WC with dyslipidemia has been variable in different studies.

Our results show that obesity was associated with lower HDL-C levels in men. These relations are similar to those described in previous studies (52-58). Some studies have shown a positive association between LDL-C and measures of adiposity (58-65), whereas other studies have failed to detect such a relationship (66-69). Another study indicated that LDL-C increased with greater abdominal circumference among younger subjects lower than 50 years (57). The Second National Health and Nutrition Examination Survey (NHANES II) data showed that excess body weight was associated with higher LDL levels in young men (62, 70).

The increase in the prevalence of obesity, abdominal obesity and body fat with age in adults has been widely documented (36, 40, 44, 50), and a similar pattern was found in our study population (Figure 5) and in Spain (22). The correlation between BMI and age and the results of the correlation analysis (Figure 4) confirmed these associations. Moreover, age was also associated with the risk of hypercholesterolemia and high LDL levels, as others have also reported (71).

Many factors affect the cholesterol levels in blood. Some of them can be controlled and others cannot. We can control what we eat, our weight, and our level of activity. However, we cannot control heredity, age, or gender. High cholesterol levels can be treated with adjustment of diet by limiting the amount of saturated fats and cholesterol that we eat. This can be achieved by following a low fat, high fibre diet. It is recommended that less than 10% of calories come from saturated fat, an average of 30% of calories or less from total fat, and less than 300 mg a day of dietary cholesterol (22). Losing weight if we are overweight can help to lower LDL-C. Regular physical activity can help raise HDL and lower LDL (72).

The present study has some limitations. The study was cross-sectional, preventing assertion of a causal relationship between BMI, WC and lipid profile. The data were sampled from only one college, so there was a possibility of selection bias and some limitation in generalization of results.
